# Comparing in-person and virtual delivery of a national ophthalmology revision course: a mixed-methods evaluation

**DOI:** 10.1186/s12909-025-08194-4

**Published:** 2025-11-27

**Authors:** Keya Jafari, E Kerr, A Seyed-Safi, N Okhravi, R G Mathew

**Affiliations:** 1 Moorfields Eye Hospital NHSFT, London, UK; 2 Directorate of Undergraduate Education, Moorfields Eye Hospital NHSFT, 162 City Road, EC1V 2PD London, UK; 3 James Paget University Hospital NHSFT, Great Yarmouth, UK; 4 University College London Hospital NHSFT, London, UK; 5 UCL Institute of Ophthalmology, London, UK; 6 NIHR Biomedical research Centre at Moorfields Eye Hospital NHSFT, London, UK

## Abstract

**Background:**

The COVID-19 pandemic prompted a rapid shift towards virtual learning. As such, the annual Duke Elder Preparatory Course pivoted to an online format using innovative teaching methods. There is a lack of prior studies directly comparing virtual to in-person delivery of the same revision course. We reviewed qualitative and quantitative student feedback across a four-year period from students who attended in-person (2019–2020, *n* = 103) and those who attended virtually (2020–2021, *n* = 215). We analysed key themes, including interactivity, attendance, and engagement, with a view to sharing key learning points with fellow educators.

**Methods:**

An integrated didactic teaching approach was used in both the in-person and virtual cohorts, with an online platform for real-time multiple-choice questions. We evaluated student feedback from 255 attendees over a four-year period using a utilisation-focused evaluation approach.

**Results:**

Global course feedback was consistently high, with a mean score of 86% for the in-person cohorts (*n* = 69) and 89% for the virtual cohorts (*n* = 186). There was a statistically significant improvement in the overall score for Content and Clarity for the virtual courses compared to the in-person courses (*p* < 0.005).

**Conclusions:**

The transition to virtual teaching can be efficient and effective for the delivery of revision courses. Students benefit from the removal of geographic barriers, the ability to interact with educators via technology-enhanced learning (TEL) and the flexibility of asynchronous virtual learning. This study highlights how educators can best utilise technological advances to improve online learning, reach larger audiences and actively monitor engagement. Virtual teaching enhanced by TEL can be used in place of in-person teaching to widen access to education and improve the student experience in large-group teaching.

**Supplementary Information:**

The online version contains supplementary material available at 10.1186/s12909-025-08194-4.

## Background

The recent shift towards virtual learning in medical education has led to increased interest in evaluating its effectiveness. While several studies suggest that online teaching was well received during the COVID-19 pandemic [1–4], challenges remain in ensuring consistent quality across different formats and contexts of virtual teaching delivery. In particular, more evidence is needed to understand how virtual delivery performs in more intensive teaching settings, such as in medical education and revision courses (intensive review courses designed to prepare students for examinations), where large volumes of content are delivered over a short period.

Virtual learning presents well-recognised challenges for both students and teachers. Students often report difficulty maintaining concentration and are subject to increased environmental distractions, which can affect learning strategies and motivation [5–7]. Teachers also face additional challenges in delivering content virtually. A lack of visual and auditory cues in a virtual setting can make it difficult for teachers to recognise when student engagement is diminishing. This, alongside limited technical competency, can lead to a loss of student-tutor interactivity [3, 8]. These challenges are particularly significant within the context of medical education, whereby a large amount of knowledge must be consumed in a short space of time in a large group setting, which can amplify the challenges involved in virtual delivery [9, 10].

Despite these challenges, virtual learning offers potential advantages such as improved accessibility, flexibility, and reduced travel or venue costs [11]. To maximise these benefits, it is important to understand when virtual teaching is appropriate and how it can be improved. Though the literature covers many aspects of virtual education, there are no studies comparing the same course delivered both in-person and online, in the context of revision courses.

We developed the Duke Elder Preparatory Course as an annual revision course to help students prepare for the national Duke Elder Undergraduate Ophthalmology Prize Examination [12]. This prestigious and highly competitive examination is taken by over 450 students annually and contributes 1–2 portfolio points for entry into UK Ophthalmology specialist training. The course was delivered in-person until 2020 and transitioned to a virtual format in 2021 and 2022 in response to the COVID-19 pandemic.

This study evaluates the impact of that transition on teaching and learning. Specifically, we examine interaction and engagement – two critical components of educational effectiveness – through both qualitative and quantitative measures across four years of course delivery. Using a Utilisation-Focused Evaluation (UFE) framework, we explore how virtual teaching may replicate or depart from the in-person experience and identify strategies to optimise virtual delivery. To our knowledge, this is the first study to directly compare outcomes from the same medical revision course taught in both virtual and face-to-face formats.

## Methods

The Duke Elder Preparatory Course was delivered in-person in 2019 and 2020 and transitioned to a virtual format for the 2021 and 2022 courses in response to the COVID-19 pandemic. The course content, structure, and course organisers remained consistent across all four years.

### Course faculty

The course was organised by a team of medical students, foundation doctors, senior ophthalmology trainees and consultants, with administrative support from the ophthalmology and medical education departments. Teaching was delivered by medical students and foundation doctors who had scored highly in the Duke Elder Examination in previous years. All teachers were mentored by ophthalmology consultants and senior registrars, who provided oversight and quality assurance. Course content and multiple-choice questions (MCQs) were mapped to the Royal College of Ophthalmologists Duke Elder curriculum.

### Course content and structure

**Fig. 1:**
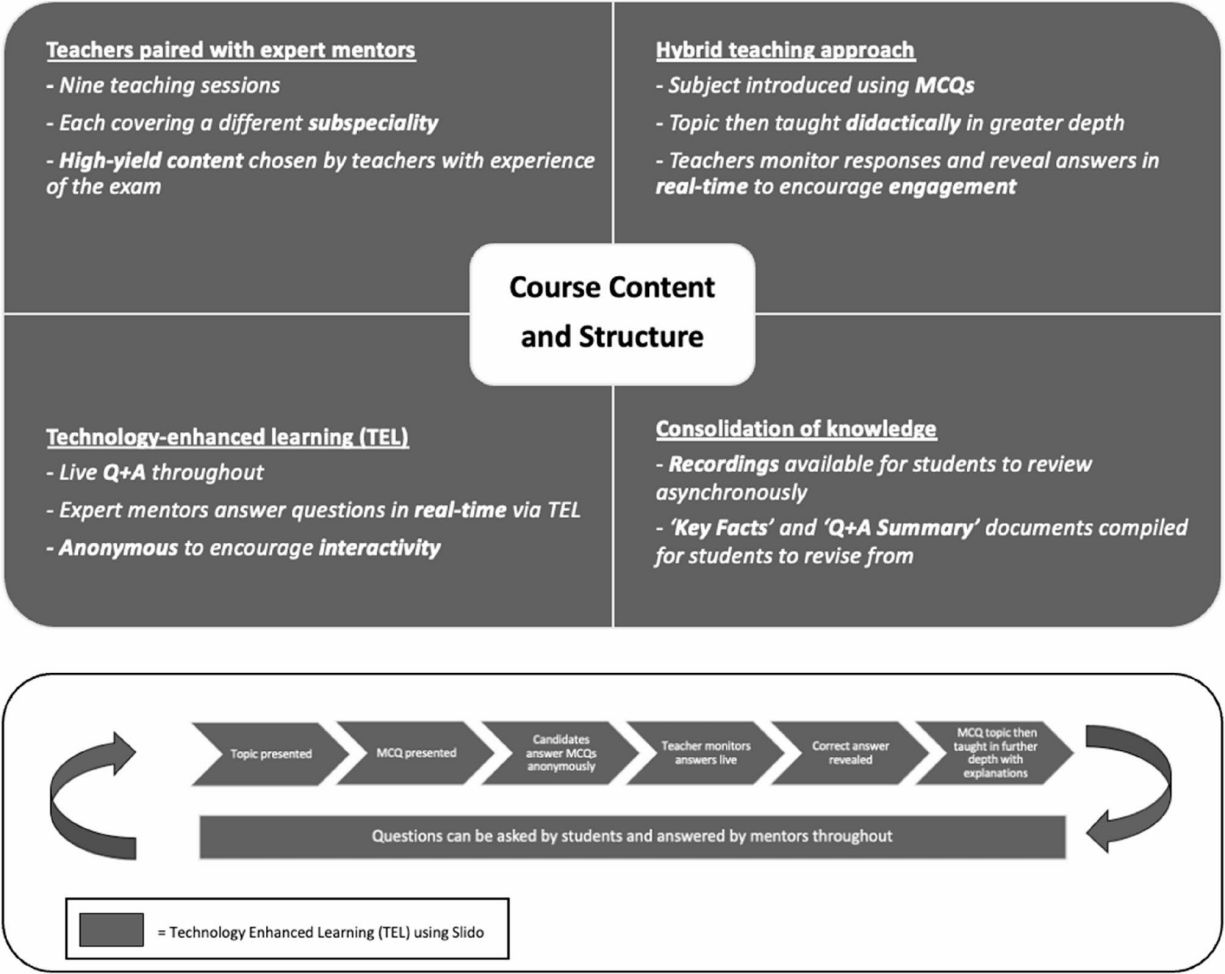
Course content and structure. Key features include a hybrid teaching approach, with MCQs interspersed with didactic teaching. TEL was used in the form of Slido to improve student engagement and efficiency of learning

Teaching was delivered through a hybrid approach, combining didactic lectures with interactive components. Technology-Enhanced Learning (TEL) was incorporated using the Slido platform (www.slido.com), which enabled real-time audience participation and anonymous submission of MCQ responses (see Fig. 1). This format was maintained in both the in-person and virtual course iterations.

### Virtual course adaptations (2021–2022)

During the transition to virtual delivery, efforts were made to preserve the core features of the course. Technical drop-in sessions were held for faculty prior to the course to ensure familiarity with the TEL platform and virtual delivery. The course timetable was adjusted to include additional breaks to minimise screen fatigue and help students maintain focus. Table 1 summarises the challenges involved in large group virtual learning and the specific adaptations made to the course design in order to overcome these challenges.

**Table 1:** Overcoming the challenges of large group online learning

Challenges of online delivery	Overcoming challenges
Clarity of teaching may be lost in the online format	• Hybrid teaching format using MCQs with didactic teaching to reinforce the teaching point. • Expert checks of slides beforehand. • Reduced word count per slide; clearly laid out slides.
Limited attention	• Mini breaks between sections were incorporated into the timetable. • MCQs were used throughout to keep people engaged.
Reduced interactivity and audience engagement	• Slido Live Q&A feature with a dedicated expert to answer questions (separate to the presenter, so as not to distract from the content presented). • MCQs throughout the talks with live response numbers encouraged audience participation.
Accessibility and technical challenges of online learning	• 1–1 Technical checks sessions ahead of the course with tutors to ensure familiarity with the chosen TEL platform (Slido). • Delegating roles for: monitoring admissions to the virtual platform, recording the sessions and keeping to time. • Setting up a separate communication channel with the course faculty and a separate group with all teachers, in order to communicate outside of the course throughout the day.
Lack of Q&A discussion	• Live Q&A feature on Slido • Encouraging questions throughout and delegating expert mentors to answer questions for each talk.

### Evaluation framework

This study used a Utilisation-Focused Evaluation (UFE) framework, which is a decision-oriented approach that prioritises the needs and intended use of the evaluation by its primary users (e.g., course organisers, medical educators) [13]. UFE focuses on producing actionable findings that can inform decisions about future course design and delivery. In this study, the framework was used to guide question formulation, data collection, and interpretation, with a focus on practical improvements to course engagement and learning effectiveness.

### Evaluation questions

The evaluation questions were developed based on known challenges in virtual teaching identified in the literature, as well as reflections from course organisers and faculty. The process by which these were created and the required resources to answer the questions are shown in Table 2. The evaluation framework is shown in Table 3.

**Table 2:** The process by which evaluation questions were created and the required information needed to answer each question

Challenge	Evaluation question	Resource required to answer this question
The course may not be as successfully received in an online format	Were students satisfied with the course overall? Did students find the content of teaching relevant?	Questionnaire after each teaching section Questionnaire at the end of the course Qualitative feedback
Clarity of teaching may be lost in the online format	Did students find the clarity of teaching equivalent or better compared to the in-person format?	Questionnaire after each teaching section Qualitative feedback
Engagement levels can be impacted in online learning	Were engagement levels maintained in the online format?	TEL data (comparing MCQ responses at the start and end of the course day)
Accessibility and technical challenges of online learning	Was the course accessible? Did the course run efficiently?	Demographics of student locations/universities Qualitative feedback
Maintaining the impact of the in-person course on summative performance The goal of the exam is to perform highly; it is a prize exam, not pass or fail	Was the impact on examination performance equivalent or better in the virtual course compared to in-person? Did attendees perform in the Top 5% nationally?	Duke Elder examination national top 20 ranking list

**Table 3:** Formative evaluation framework using the UFE model

Context	The Duke Elder Revision course has been running as an online format for 2 years since 2020 in response to the COVID-19 pandemic	
Challenge	To provide evidence of large-group virtual course effectiveness and impact on learning in comparison to its previous in-person iteration	
Evaluand	1-day online course, synchronous delivery with asynchronous learning through post-course materials	
Nature	Well-established course materials that are primarily fixed, with potential for modifications for future iterations	
Type of evaluation	Formative evaluation	
Purpose of evaluation	To determine if the course meets its objectives and is as successful and effective as the in-person iteration.	
Evaluation questions	See Table 1a.	
Sources of evaluation data	Student feedback questionnaire Qualitative feedback Engagement data from TEL Accessibility data from course attendee demographics Duke Elder examination top 20 ranking	
Utility of findings	Informs modifications for future courses Supports ongoing virtual course format	

### Participants and sampling

Participants were all students currently studying medicine at a university globally. Pre-existing specialist ophthalmology knowledge was not a prerequisite, however, given this was a revision course close to the exam date, a certain level of background knowledge would have been expected from attendees. There were no exclusion criteria. It was expected that participants attend all sessions and partial attendance only occurred in limited circumstances. Participation in feedback was voluntary and anonymised, and no background information about respondents was collected. The majority of attendees were senior UK medical students (years 4–6) preparing for the Duke Elder examination. Students’ prior experience with online learning was not formally quantified. However, as the first virtual course was delivered in February 2021, most students were likely to have had up to one year of exposure to online teaching following the first UK national lockdown in March 2020. All submitted responses were included in the analysis.

There was no random sampling, and the cohort reflects a self-selecting group of students with an interest in ophthalmology who voluntarily registered for the course. This may introduce self-selection bias. However, the course consistently attracts students from a wider range of medical schools and has historically high uptake from those intending to apply for ophthalmology training.

### Data collection and analysis

#### Quantitative data

Students completed a feedback form after each taught topic. The feedback form was developed for this course, and a copy of this can be found in the supplementary materials. Included in the feedback for each section was a Clarity score, reflecting the ease with which the students were able to understand the topics, and a Content score, describing how relevant the content was for exam preparation. Clarity and Content scores were rated on a 6-point Likert scale (1 = “very poor” to 6 = “excellent”). At the end of the course day, students also completed a ‘Global Feedback’ questionnaire, providing an overall rating of the course. And free-text responses.

Student engagement during the live sessions was measured using Slido data on MCQ responses during the course. The number of MCQ responses submitted at the beginning and end of each course day was compared as a proxy for sustained engagement.

Quantitative data for 255 returned questionnaires were analysed using a two-way ANOVA using the statistical software GraphPad Prism 9 for Windows (GraphPad Software, Boston, Massachusetts, USA, www.graphpad.com).

#### Qualitative data

Open-ended responses from the Global Feedback form were analysed using reflexive thematic analysis [14]. Feedback from the virtual cohorts (2021 and 2022) was reviewed to explore student perspectives on engagement, interactivity, and overall course quality.

A qualitative consensus-building process was used to analyse the data. Each qualitative analyst independently reviewed the raw data, documenting key codes and themes. The team met to discuss their initial interpretation of the data, discussing points of agreement and different perspectives. From this analysis, three key themes were revealed: accessibility, efficiency, and engagement. TEL was identified as a unifying feature throughout. This qualitative feedback is summarised in Fig. 5. No qualitative data analysis software was used.

### Ethical considerations

As this was a service evaluation of an educational intervention with no pre-determined standards, this study was deemed exempt from ethics approval. Advice was sought from UCL’s Research Ethics Committee, who confirmed that ethics approval was not required for this study and that the need to consent to participate was deemed unnecessary. Feedback was acquired anonymously on an entirely voluntary basis as part of a service evaluation. The evaluation did not involve any changes to the service being delivered for the purpose of the review, and the change to course delivery was made in response to the COVID-19 pandemic limiting in-person teaching.

## Results

Over the four years, 318 students attended the course. A total of 103 students attended in-person (in 2019 and 2020) and 215 attended virtually (in 2021 and 2022). The number of students attending the course more than doubled between the in-person and virtual courses. A total of 255 students returned the feedback questionnaire. Response rates were high across both delivery formats, with 66.7% of the in-person cohort (2019–2020) and 86.5% of the virtual cohort (2021–2022) completing feedback questionnaires.

### Questionnaire

**Fig. 2:**
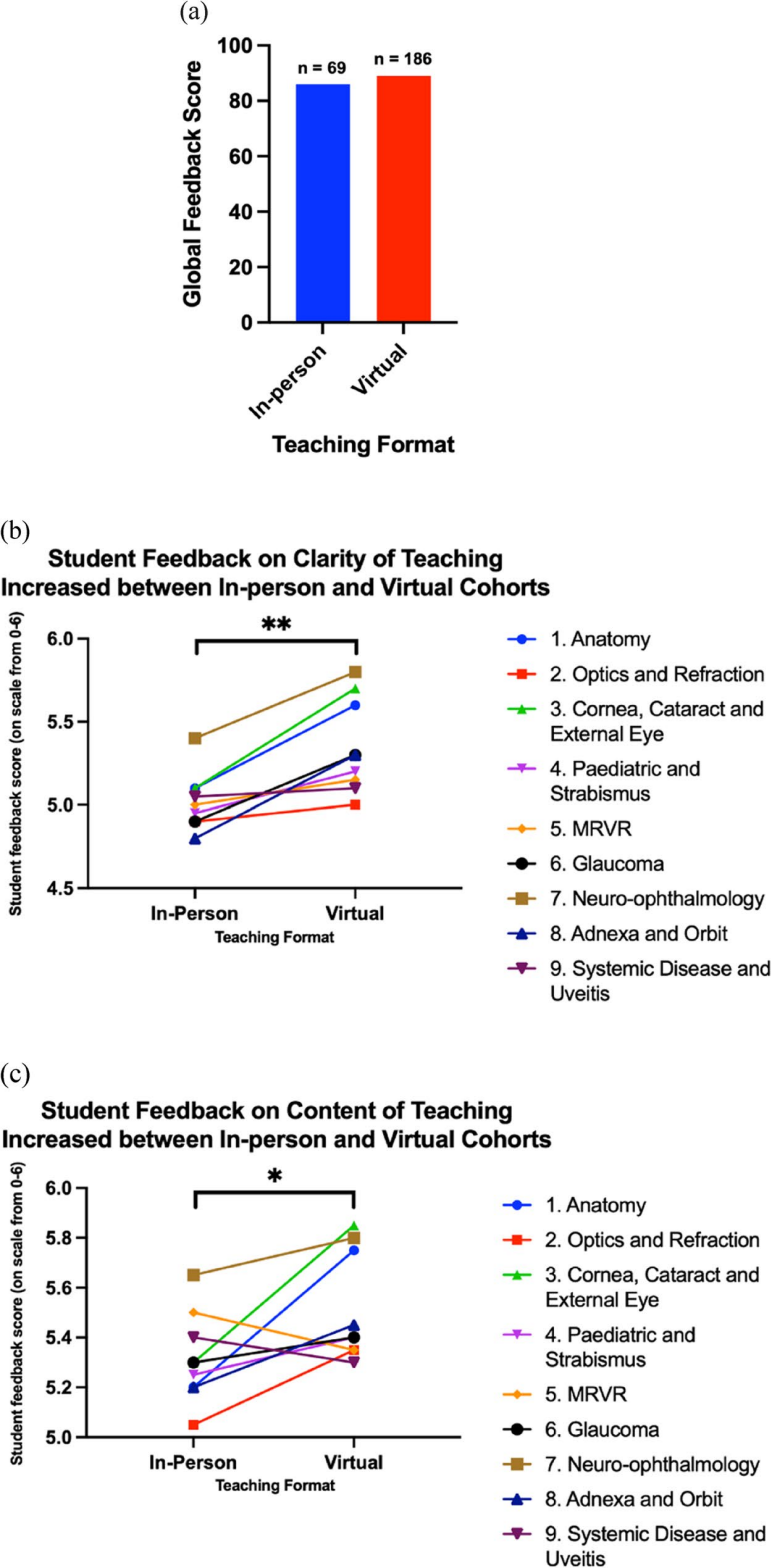
Global Feedback and Clarity and Content Scores for In-Person and Virtual Cohorts. (**a**) Mean Global Feedback Scores (0–100) from attendees of the in-person (2019 and 2020; blue; *n* = 69) and virtual (2020 and 2021; Red; *n* = 186) Duke Elder Revision courses; n = number of individual students. (**b**) Mean Clarity scores and, (**c**) Content scores for each section of the revision course, comparing in-person, (*n* = 372) and virtual, (*n* = 589) cohort responses; n = total number of topic-specific feedback responses across all students. **p < 0.005* *p* < 0.05

Overall course feedback was consistently high, with a mean Global Feedback score of 86% for in-person delivery and 89% for the virtual format (Fig. 2a).

Clarity and Content scores improved when the course transitioned from an in-person to a virtual format (Fig. 2b and c). A two-way ANOVA was conducted with Delivery Format (in-person vs. virtual) and Course Section (nine topics) as fixed factors. No significant interaction was observed between format and section. The mean Clarity score increased from 5.02 in the in-person cohorts to 5.35 in the virtual cohorts. This difference was statistically significant (*p* = 0.0011 for the main effect of format), with a mean difference of 0.33 points (95% CI: 0.18 to 0.48). The corresponding partial eta squared was 0.76, indicating a very large effect size. Similarly, the mean Content score increased from 5.32 to 5.52. This difference was also statistically significant (*p* = 0.041 for the main effect of format), with a mean difference of 0.20 points (95% CI: 0.01 to 0.39) and a partial eta squared of 0.43, reflecting a large effect.

### TEL data

**Fig. 3:**
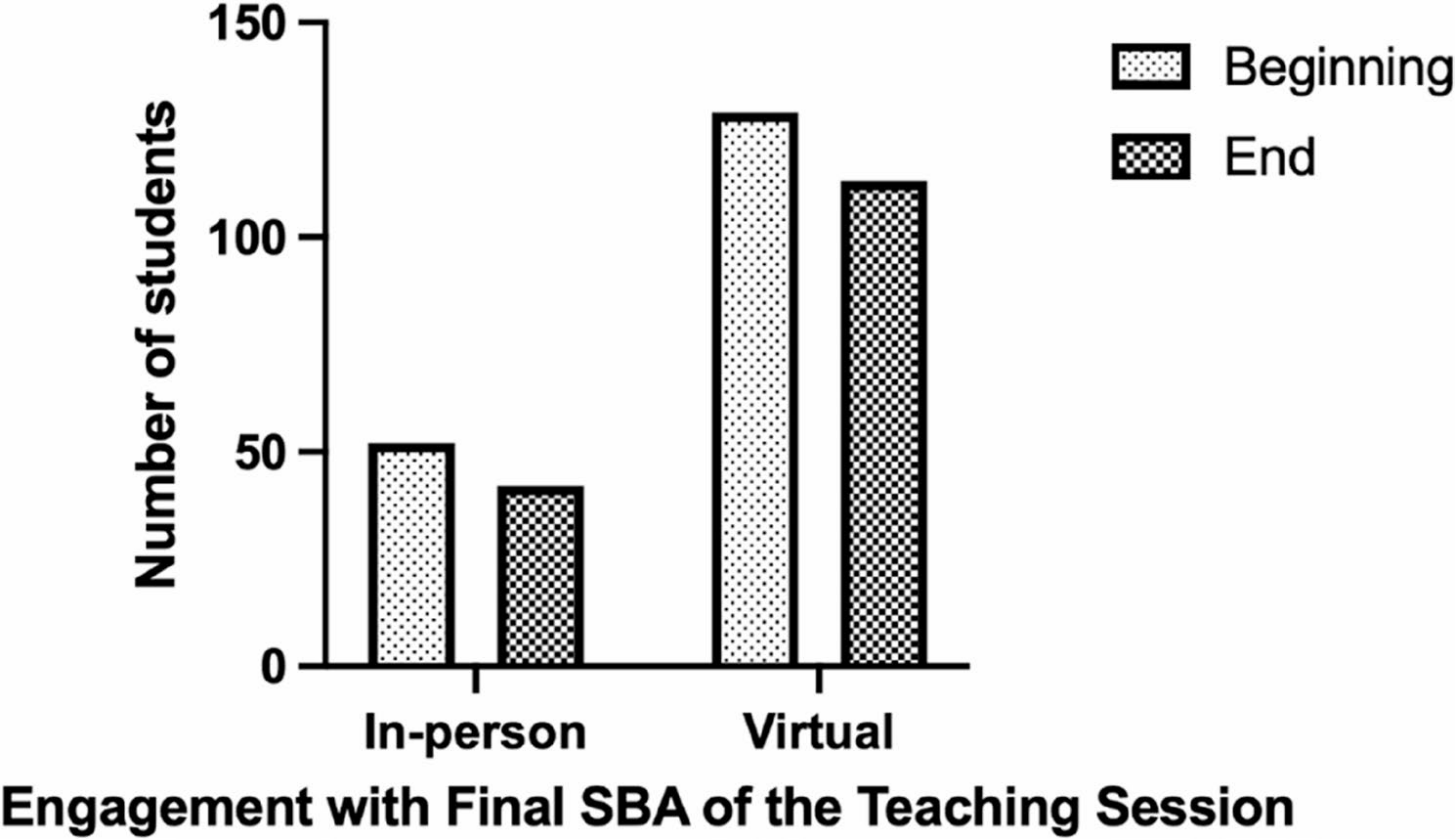
Number of Students Engaging with the MCQs at the Beginning and End of the Course in the In-Person and Virtual Cohorts. Proportion of students responding to MCQs via the TEL platform (Slido) at the beginning and end of the course. The “beginning” and “end” were defined by the final MCQ of the second and penultimate sections, respectively. Engagement was maintained throughout, with 81% of in-person and 88% of virtual attendees still responding at the end of the course

Levels of engagement were sustained throughout the course in both in-person and virtual formats (Fig. 3). By the end of the course day, 88% of students in the virtual cohorts were still participating in answering MCQs, compared to 81% in the in-person cohorts – despite the larger size of the virtual group.

### Geographic reach

**Fig. 4:**
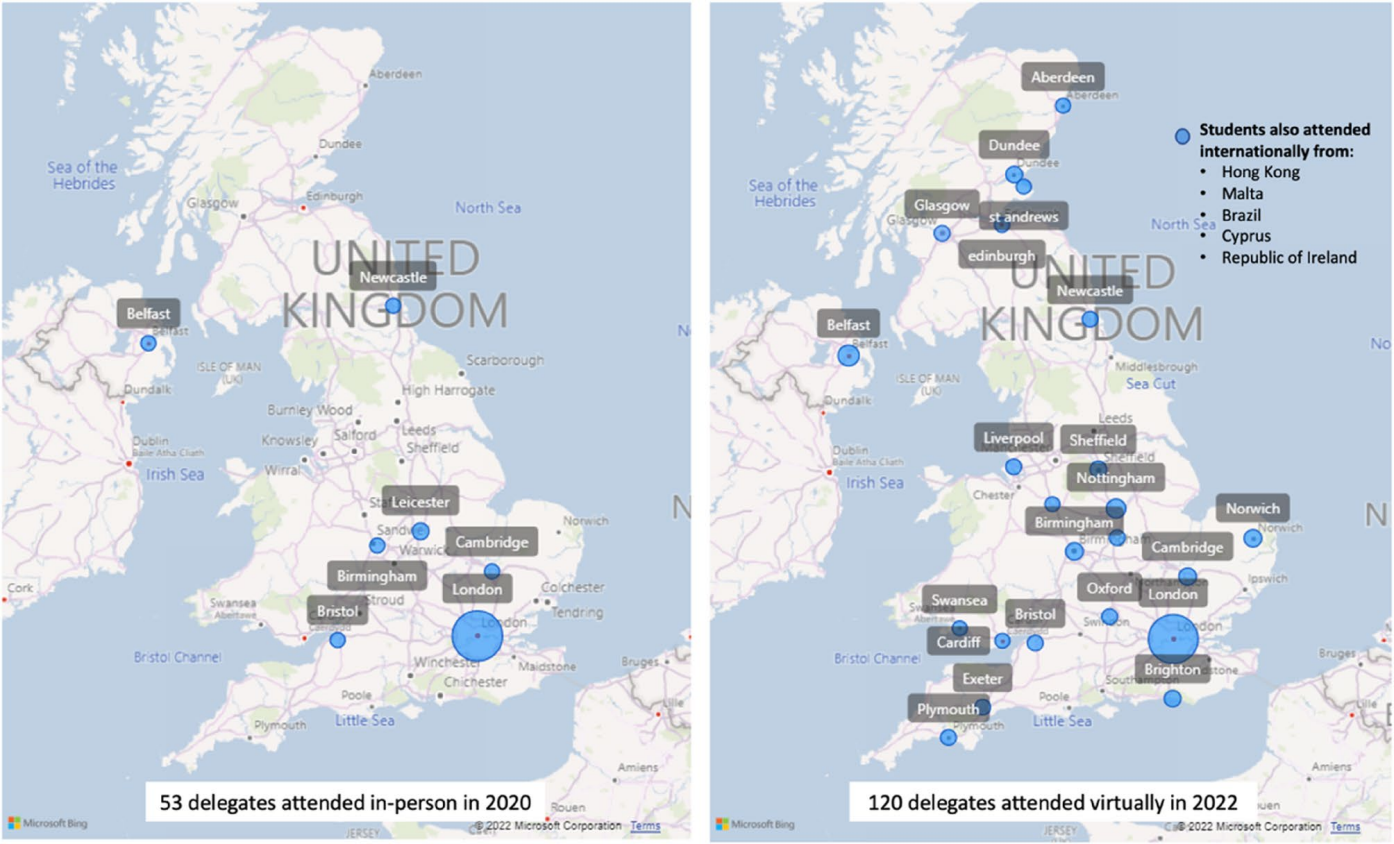
Geographic Distribution of Course Attendees in 2020 (in-person) vs. 2022 (virtual). Geographic distribution of course attendees in the 2020 in-person cohort (left; *n* = 53) and the 2022 virtual cohort (right; *n* = 120). Circle size represents the number of students from each region. The virtual format enabled broader national and international participation

Delegate university location data revealed a broader national reach following the transition to a virtual format. Figure 4 illustrates the increased diversity of delegate locations between 2020 (in-person) and 2022 (virtual). Students from 7 regions accessed the course in 2020, compared to 26 regions in 2022. This included five international participants in 2022 who attended from the Republic of Ireland, Malta, Cyprus, Hong Kong and Brazil.

#### Qualitative feedback

**Fig. 5:**
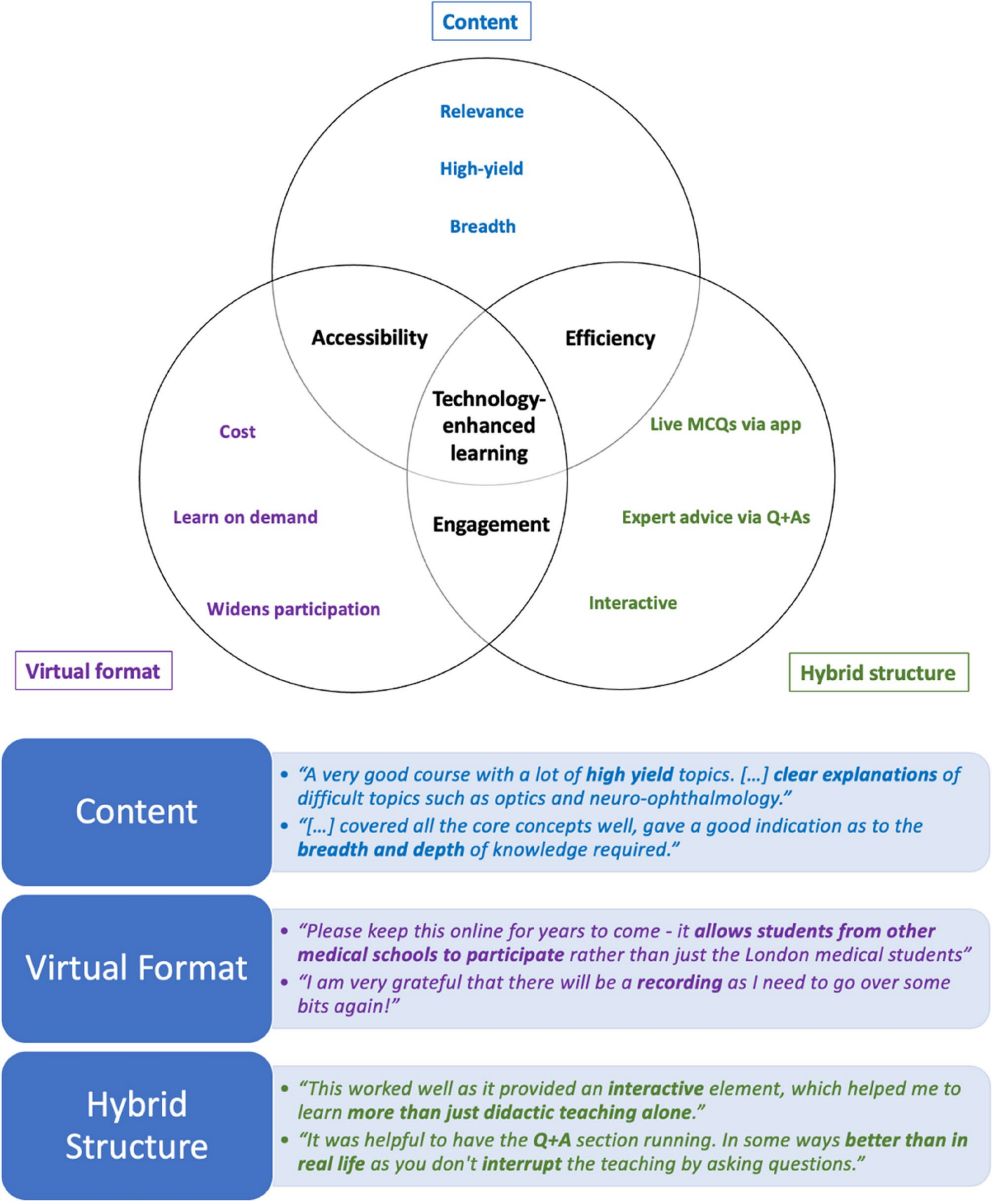
Qualitative analysis of key themes from student feedback. Concept map and table summarising key themes from thematic analysis of qualitative feedback including quotes from participants. Direct student quotes illustrate the themes of Accessibility, Efficiency, and Engagement, with Technology-Enhanced Learning (TEL) emerging as a unifying enabler

Thematic analysis of free-text responses identified three recurring themes: accessibility, efficiency, and engagement. TEL was consistently referenced as a key enabler across these themes. A summary of representative student quotes and theme mapping is provided in Fig. 5.

### Performance in Duke elder summative examination

A review of national results showed that 25.6% of the top 20-ranked candidates in the Duke Elder examination had attended the in-person course. This figure rose to 37.8% for the virtual cohorts. While not directly comparable due to potential confounders, this data suggests the virtual format maintained, if not improved, the course’s positive impact on examination performance.

## Discussion

One of the biggest challenges in online teaching is maintaining interactivity and engagement [9, 10]. This study aimed to evaluate whether virtual teaching can be as effective as in-person teaching for large-scale revision courses, using both subjective and objective measures. The outcomes demonstrate that virtual delivery can maintain – and in some aspects, improve – key educational outcomes, particularly when underpinned by Technology-Enhanced Learning (TEL).

Thematic analysis of the qualitative feedback highlighted three key themes of engagement, efficiency and accessibility. TEL was central to the development of these features in the virtual format. Our results provide strong support for the use of TEL to maintain interactivity and engagement in virtual teaching for large audiences.

### Engagement and learning outcomes

The ‘Global Feedback’ score remained consistently high for the virtual format, with a mean score of 89% compared to 86% for the in-person cohorts (Fig. 2a). Moreover, there were statistically significant improvements in both Clarity and Content scores for the virtual cohorts, suggesting that virtual delivery did not compromise and may have enhanced students’ perception of learning quality.

The objective measures also support this. Engagement, as measured by MCQ participation, was comparable across course formats, with 88% of students still responding at the end of the virtual course compared to 81% in the in-person cohort. This sustained engagement occurred despite a larger class size in the virtual format (mean of 108 virtual attendees vs. 52 in-person attendees per course), highlighting the utility of TEL in promoting interactivity.

TEL features such as anonymous Q&A and live MCQ polling supported student participation and allowed teachers to tailor content dynamically based on live feedback. These findings align with the *Community of Inquiry* framework [15], which emphasises cognitive presence (engagement with content), social presence (interaction with peers and faculty), and teaching presence (facilitation by instructors). TEL features such as live MCQ polling and anonymous Q&A supported all three elements simultaneously, fostering an environment more consistent with the principles of a CoI. During virtual teaching, teachers ordinarily have difficulty monitoring engagement due to the lack of visual and auditory cues. Live data via TEL enabled instant feedback on engagement levels for teachers. This allowed teachers to view the proportion of students answering MCQs in real time, as well as the proportion of students answering correctly, allowing them to encourage students to participate if response rates were low and to shift focus to more challenging content.

Additionally, anonymity is an important consideration in promoting engagement and participation in large-group settings [16]. Qualitative data reflected that the anonymous nature of asking and answering questions virtually using TEL encouraged participants to engage and submit questions during the course (Fig. 5).

### Efficiency and cognitive load

The Duke Elder Preparatory Course encompasses the challenge of presenting a vast array of complex subspeciality topics using unfamiliar terminology in a short space of time. We believe the hybrid MCQ-didactic format used in both virtual and in-person iterations enhances learning through several mechanisms. The Segmenting Principle from Mayer’s *Cognitive Theory of Multimedia Learning* suggests that learners benefit when information is presented in manageable segments with pauses for processing [17]. By interspersing MCQs between teaching segments, the course may reduce cognitive overload by breaking complex topics into manageable chunks, allowing students to pause, process, and consolidate before moving on. Furthermore, this structure - alongside TEL delivery - draws on the principles of *Test-Enhanced Learning*, which provides strong evidence that retrieval practice, such as answering MCQs, improves learning and long-term retention [18, 19]. Rather than passively receiving information over a virtual platform, students engaged in repeated cycles of retrieval and feedback, helping to reinforce key concepts throughout the course. From the perspective of *Cognitive Load Theory (Sweller)*, the hybrid MCQ–didactic format likely reduced extraneous cognitive load by breaking down complex material and guiding attention to key concepts, while promoting germane load through retrieval practice and feedback [20, 21].

Additionally, the time constraints of a revision course leave limited opportunity for attendees to ask questions after each talk. In our teaching model, the TEL platform allowed for questions to be submitted and responded to by the expert mentors simultaneously during the talks. An average of 141 distinct questions were responded to per virtual course, a number that would not have been feasible without the use of TEL.

### Access and equity

Thematic analysis highlighted a perceived increase in accessibility in the virtual format. Students noted that removing geographic and financial barriers enabled broader participation (*“allows students from other medical schools to participate rather than just the London medical schools”)*, particularly for those at institutions without formal ophthalmology teaching (Fig. 5).

### Impact

The impact of our courses was assessed by reviewing the number of students who achieved a top 20 ranking in the national Duke Elder examination. This cut-off was chosen as the top 20 candidates receive a special commendation and 2 points for speciality training applications (with all other passing candidates receiving 1 point). The in-person course successfully achieved this, with 25.6% of the candidates in the top 20 list having attended the course. For the virtual course, 37.8% of the top 20 scores were obtained by virtual course attendees.

While promising, this finding should be interpreted cautiously and is not suitable for direct comparison. Firstly, this represents a select subset of outcomes from the exam and cannot be generalised to all course attendees’ performance. Additionally, the increase in the proportion of top-scoring candidates in virtual cohorts has multiple confounding factors, including the increased accessibility and number of course attendees (2-fold increase in virtual cohorts), as well as iterative improvements in the course content and design through annual review of student feedback. Nonetheless, it does clearly demonstrate that the virtual delivery of the course did not have a detrimental impact on the attainment of attendees.

### Limitations

#### Methodological limitations

Questionnaire-based feedback is inherently subject to self-report and response biases. Although anonymity was assured to mitigate this, the potential for positive skew remains. Additionally, feedback was collected using structured forms rather than interviews or focus groups, limiting the depth of qualitative insights. However, the sample size was sufficient to identify recurring themes across cohorts.

Evaluator bias is another important consideration, as all authors were involved in both course delivery and evaluation. While quantitative measures were predefined and thematic analyses were conducted collaboratively to reduce subjectivity, some degree of bias cannot be excluded.

#### Interpretive limitations

Although most teachers from the in-person course also contributed to the virtual versions, the course content and delivery methods evolved year on year. Teachers naturally refined their materials based on previous feedback and experience, which may have independently influenced student ratings. Likewise, students in later cohorts may have benefited from general improvements in course structure unrelated to the format of delivery.

Students’ prior exposure to online learning was not formally assessed. However, given the timing of the virtual courses during the COVID-19 pandemic, it is likely that participants had at least one year of experience with remote learning. This increased familiarity may have influenced both engagement and satisfaction levels. Additionally, the reduction of teaching events across universities globally during COVID-19 may have contributed to increased engagement of participants in the virtual courses.

#### Generalisability limitations

The findings of this study are based on a single ophthalmology revision course with self-selecting attendees, many of whom were highly motivated and preparing for a competitive national examination. As such, the results may not be generalisable to all medical students or to courses with different formats, content, or learning objectives.

#### Future directions

Long-term knowledge retention and clinical impact were not evaluated. To address this, future iterations of the course will incorporate follow-up assessments and focus group discussions to gain deeper qualitative feedback and assess sustained learning outcomes.

## Conclusions

This evaluation demonstrates that large-scale in-person revision courses can be successfully adapted to a virtual format without compromising educational quality. Technology-Enhanced Learning (TEL) played a central role in supporting interactivity, engagement, and efficiency. Both subjective and objective outcomes suggest that virtual delivery preserved – and in some areas improved – the learning experience. Importantly, accessibility was broadened without sacrificing engagement metrics. This study offers a replicable model for transitioning large group teaching to virtual platforms in a pedagogically robust way, widening the reach of these findings beyond medical revision courses.

## Supplementary Information

Supplementary Material 1.

## Data Availability

The quantitative and qualitative data used to support the findings of this study are available from the corresponding author upon request.
