# Supplementary material for: Comparing in-person and virtual delivery of a national ophthalmology revision course: a mixed-methods evaluation
Source: BMC Med Educ. 2025 Nov 27;25:1661. doi: 10.1186/s12909-025-08194-4 (PMC12659102; doi:10.1186/s12909-025-08194-4)
Supplement: Supplementary file 1 — Supplementary Material 1. [file 12909_2025_8194_MOESM1_ESM.docx]

# Duke Elder Prep Course, February 8^th^, 2020

# Evaluation and Feedback

This feedback is anonymous

# Please circle the appropriate number:

# 1 = Very poor 2 = poor 3 = satisfactory 4 = good 5 = very good 6 = excellent

| **Can you tell us what you think of the mixed format (virtual classroom and didactic lecture) of this course?** |  |
| --- | --- |

| **Speakers** | Presentation | **Content** | Comments |
| --- | --- | --- | --- |
| **[name redacted]**  Anatomy, Physiology & Epidemiology | 1 2 3 4 5 6 | 1 2 3 4 5 6 |  |
| **[name redacted]**  Glaucoma | 1 2 3 4 5 6 | 1 2 3 4 5 6 |  |
| **[name redacted]**  Cataract, Cornea & External Eye | 1 2 3 4 5 6 | 1 2 3 4 5 6 |  |
| **[name redacted]**  Paediatrics and Strabismus | 1 2 3 4 5 6 | 1 2 3 4 5 6 |  |
| **[name redacted]**  Medical Retina and VR | 1 2 3 4 5 6 | 1 2 3 4 5 6 |  |
| **[name redacted]**  Refraction and Optics | 1 2 3 4 5 6 | 1 2 3 4 5 6 |  |
| **[name redacted]**  Neuro ophthalmology | 1 2 3 4 5 6 | 1 2 3 4 5 6 |  |
| **[name redacted]**  Adnexal and Orbital | 1 2 3 4 5 6 | 1 2 3 4 5 6 |  |
| **[name redacted]**  Systemic Disease and Uveitis | 1 2 3 4 5 6 | 1 2 3 4 5 6 |  |
| **Virtual Classroom App** | **Ease of use**  1 2 3 4 5 6 | **Facilitating learning**  1 2 3 4 5 6 | **Would you like to use the app again at another course? Yes / No / Maybe** |
| **How helpful was this course for your learning?** | | 1 2 3 4 5 6 | |

| On a scale of 0-100 (0 expectations were not met- 100 expectations were exceeded) how would you rate the course? | My score is: |
| --- | --- |

*Thank you for filling out our evaluation form*
